# 

**Published:** 1915-02

**Authors:** 


### CONTENTS.

ORIGINAL COMMUNICATIONS—

Pellagra—Preliminary Paper, by Rufus T. Dorsey, M. D. .......................................499

Case of Hodgkins Disease, by L. C. Rouglin, M. D. .......................................506

Meeting of the Fulton County Medical Society, by R. R. Daly, M. D. ............................510

EDITORIAL .......................................515

SELECTIONS AND ABSTRACTS ..................517

BOOK REVIEWS ..............................554

